# Roles of Hardened Husks and Membranes Surrounding *Brachypodium hybridum* Grains on Germination and Seedling Growth

**DOI:** 10.3390/plants8090322

**Published:** 2019-09-03

**Authors:** Ali El-Keblawy, Masarra Elgabra, Kareem A. Mosa, Amal Fakhry, Sameh Soliman

**Affiliations:** 1 Department of Applied Biology, College of Sciences, University of Sharjah, Sharjah PO Box 27272, UAE (M.E.) (K.A.M.); 2 Department of Biotechnology, Faculty of Agriculture, Al-Azhar University, Cairo 11651, Egypt; 3 Department of Botany and Microbiology, Faculty of Science, Alexandria University, Alexandria 21568, Egypt; 4 Department of Medicinal Chemistry, College of Pharmacy, University of Sharjah, Sharjah PO Box 27272, UAE

**Keywords:** *Brachypodium distachyon* complex, dormancy regulating compounds, grain dormancy, grasses, hardened husks, light quality

## Abstract

Several studies have assessed the function and significance of the presence of dead, hardened husks on germination and seedling growth in several grass species and reached to inconsistent results. Here, we assess the roles of husks (dead lemma and palea) and an inner membrane surrounding the grains on germination behaviour and seedling growth of *Brachypodium hybridum*, one of three species of the genetic model *B. distachyon* complex, in an arid mountain of Arabia. The interactive effects between temperature and the incubation light were assessed on germination of husked and dehusked-demembraned grains. Germination and seedling growth were assessed for different combinations of grain treatments (soaked and non-soaked husked, dehusked-membraned and dehusked-demembraned). Dehusked-demembraned grains were also germinated in different dormancy regulating compounds (DRCs) and light qualities (light, dark and different red: far red [R: FR] ratios). The results indicated an insignificant difference between husked and dehusked-membraned grains on final germination and the germination rate index (GRI), with the former producing significantly bigger seedlings. Removal of the inner-membrane resulted in a significant reduction in all traits. Soaking grains in water resulted in significant enhancements in germination and seedling growth of only husked grains. Husked-membraned and demembraned grains germinated more significantly and faster at lower rather than higher temperatures. None of different concentrations of several DRCs succeeded in enhancing final germination of dehusked-demembraned grains. Red-rich light significantly enhanced germination of dehusked-membraned grains in comparison to other light qualities. It could be concluded that the role of husks is to mainly enhance seedling growth, while the major role of the membrane is to increase final germination. The ability of red-rich light in enhancing the germination of dehusked-membraned but not dehusked-demembraned grains suggest a role for the inner membrane in regulating dormancy through differential filtering of light properties.

## 1. Introduction

Grasses have evolved modified inflorescence structures around flowers comprised of lemma (external structures) and palea (internal structures) [1]. It has been assumed that dead structures enclosing embryos (husk) provide physical protection and help in seed dispersal [1]. In addition, these dead structures can potentially protect fruits from predation, position and anchor fruits in the soil as well as absorb moisture to stimulate germination [2]. Recently, Raviv et al. reviewed the biochemical activities of dead structures enclosing the fruits of several plant species belonging to different families, including Poaceae, and concluded that these structures contain various active enzymes involved in the hydrolysis process (e.g., nucleases, proteases, and chitinases) and detoxification of reactive oxygen species [3]. Such enzymes can control seed germination and enhance growth of germinated seedlings [3,4]. In addition, dead structures around fruits of *Arabidopsis thaliana* and *Sinapis alba* had active hydrolytic enzymes that can be released upon hydrolyses to increase the survival rate of emerged seedlings [5]. The same authors indicated that the dead structures enclosing fruits could provide the embryo with a nutritional element, such as nitrate, potassium, phosphorus and sulfur. Such nutrients and metabolites have the potential to support the embryo during storage in the soil, control germination and enhance seedling establishment [3,5].

Several studies have reported the importance of husk enclosing grains of grasses in the enhancement of the germination process [6,7]. For example, external structures around the grains of *Festuca* caryopsis increased both final germination and salt tolerance [7]. Similarly, germination in *Lolium perenne* was higher for grains sown in husks in comparison to naked grains [6]. However, in other grasses, husks induced grain dormancy. In wild emmer wheat, for example, intact dispersal units hindered grain germination (i.e. induced dormancy) but enhanced seedling growth, especially in the root system [8]. In addition, the presence of the husk around the caryopses of *Aegilops kotschyi* inhibited germination [9]. Furthermore, in other species of family Asteraceae, the pericarp induced seed dormancy as it acted as a mechanical constrain hampering water uptake in *Glebionis coronaria* [10] and oxygen availability in *Helianthus annuus* [11]. In many of the species where germination was inhibited because of husk presence, a significant increase in germination was observed after husk removal. This would indicate that the dormancy associated with husk presence is a physical rather than embryo-based dormancy [4,9,12]. Despite the roles of husk involvements in final germination having received considerable investigation, little is known about the possible husk mechanisms linked to light control requirements during germination.

Seed dormancy is a strategy adopted by plants living in unpredictable environments to delay seedling emergence until the arrival of conditions favorable for survival and growth [13]. Therefore, germination percentage and speed are amongst the most sensitive life history traits that could affect the fate and fitness of the emerged seedlings [14]. Seed germination and dormancy are modulated by complex interactions between dead structures surrounding embryos [3,4,8] and post-dispersal conditions prevailing during seed storage and soaking [15,16,17]. For example, maternal plants control seed traits through their contribution to cell organelles, seed coat structure, quantity and quality of endosperm, as well as provisioning metabolisms such as proteins, nutrients and phytohormones. All of these factors can affect seed dormancy and seedling establishment [18]. Additionally, dormancy and germination responses are controlled by conditions prevailing during seed soaking [19,20]. Therefore, the timing of seed germination is controlled by individual or multiple factors including structures surrounding the embryo, the maternal environment, storage conditions, and the suitability of environmental conditions during seed soaking.

Dormancy regulating compounds such as phytohormones (e.g., gibberellic acid, abscisic acid, kinetin and ethylene) and nitrogenous compounds (e.g., thiourea and nitrate) have been reported to regulate germination and break the innate dormancy of several species of dry subtropical deserts [21,22,23]. For example, some studies indicated the presence of several phytohormones in dead structures enclosing the embryo with the capacity to regulate seed germination and seedling growth. For example, the glumes of wild emmer wheat contain abscisic acid (ABA) that enhance dormancy and promote tolerance against biotic and abiotic stresses in the emerged seedlings, as well as growth promoting hormones, such as auxin, jasmonic acid and salicylic acid that can enhance germination, and seedling growth and development [8]. In addition, the exogenous application of nitrate and thiourea alleviated innate dormancy in seeds of several grasses including *Sporobolus arabicus* [24], *Lasiurus scindicus* [25], *Centropodia forsskalii* and *Sporobolus spicatus* [21], as well as *Aristida adscensionis*, *Eragrostis ciliaris* and *Tragus racemosus* [26]. Other studies [23,27] reported that nitrogenous compounds could terminate seed dormancy by enhancing levels of cytokinin and reducing levels of ABA, which is associated with enhancing innate dormancy. In addition, nitrate can act as a hormone that regulates the germination process through phytochrome [28]. Similarly, exogenous application of GA and kinetin ended dormancy in certain grasses including *Eragrostis ciliaris* [26].

The genus *Brachypodium* is a grass (family Poaceae) native to the Mediterranean region, with accessions recorded in southern Europe, North Africa and Eurasia [29]. However, the introduced range of Brachypodium covers all six continents [30]. *Brachypodium distachyon* exists as a species complex of three taxa (*B. distachyon* with 2n = 10 chromosomes, *B. stacei* with 2n = 20 chromosomes, and *B. hybridum* with 2n = 30 chromosomes) [31] *B. distachyon* and its two close relatives (*B. stacei* and *B. hybridum*) have been proposed as valuable genetic models for grain studies at molecular, physiological and ecological levels [31]. In addition, the *Brachypodium* complex has been considered as a model for pasture and bioenergy crops [31,32]. The three *Brachypodium* species are native to the Mediterranean region, with *B. hybridum* being the only species that is known to be exotic in other parts of the world (e.g., California, Australia, South America and South Africa) [33]. The three species cannot be distinguished based on their morphological features [33,34]. In the hot arid Arabia, *Brachypodium* has been recorded as a native species in the Hajar Mountains and defined, based on morphological features, as *B. distachyon* [35,36]. However, using microsatellite SSR (single sequence repeats) analysis with four different markers (ALB165, ALB311, BdSSR330, and R2-3-ABI), the species in the United Arab Emirates (UAE) has been defined as *B. hybridum* (Masarra Elgabra, Kareem Mosa, Abdelaziz Tlili and Ali El-Keblawy, in preparation).

**Figure 1:**
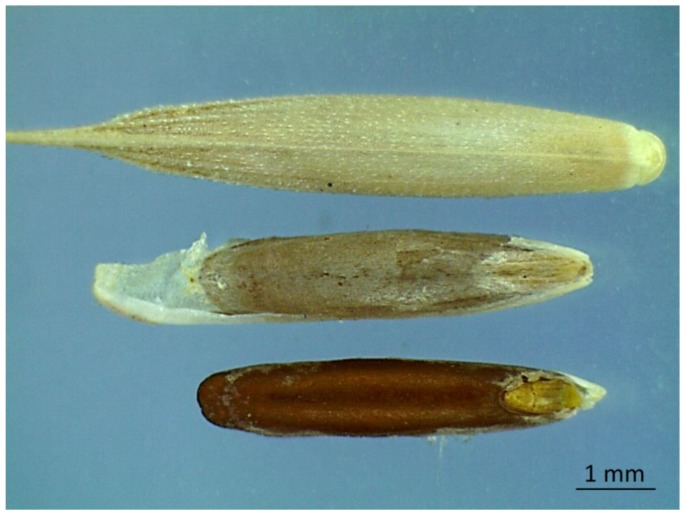
The difference between husked, dehusked with membrane and dehusked without inner membrane grains of *Brachypodium hybridum* (at 20*0.65 = 13 x magnification under stereomicroscope).

The anatomy of the embryo of *Brachypodium* is almost identical to that of cereals, such as barley and wheat. This embryo is a single cotyledon with apical meristem that is enclosed by a coleorhiza and coleoptiles [37]. In addition, the overall seed size and external anatomy of mature grains of the diploid *B. distachyon* are very similar to those of the grains of major cereals such as rice and wheat, with the exception of the smaller endosperm volume of the former [37]. *Brachypodium* has been considered as a good model plant for studying grain dormancy of cereal crops [12]. Additionally, *Brachypodium* grains are covered by a husk (lemma and palea), a structure present in most wild grasses. Barrero et al. reported the inhibition of germination in grains covered with husk structures in the genetic model grass *B. distachyon* [12]. The authors indicated that grain dormancy was relieved with manual removal of husk structures [12]. Our investigation of *B. hybridum* grains indicated the presence of a whitish inner membrane (exocarp) covering them (Figure 1).

The investigated dead structures having roles in germination and seedling growth included seed coats, pericarps and floral bracts in grasses [3,4,5]. According to our knowledge, the only study that assessed the role of an inner membrane enclosing the embryo was that of Koller et al. [38] in *Citrullus colocynthis*, a dicot species. The innate dormancy of the seeds of this species was alleviated by the removal of the inner membrane [38]. In our study, we assume possible roles for both the husk and the inner membrane on grain germination and seedling growth of *B. hybridum*. This species has been domesticated and selected commercially as a suitable cover crop grass to protect olive groves, vineyards and dry fruit croplands [39,40]. In addition, *B. hybridum* was proposed as a promising soil cover for hillside and steep vineyards [41]. Selecting grain characteristics that would result in higher germination and faster growth is important for this cover crop grass. Therefore, the aim of the present study was to assess the impact of the surrounding structures (husk and the inner membrane) on seed germination and growth of *B. hybridum* seedlings. As the dead surrounding structure might affect light filtering properties and the production of dormancy regulating compounds, the study also aimed at assessing the effect of light quality (i.e., different R:FR ratios) and exogenous application of different dormancy regulating compounds on final germination, germination speed and seedling growth. 

## 2. Results

### 2.1. Effects of Husk Treatments on Germination

Husk treatments had significant effects on final germination (F = 58.5, *p* < 0.001) and germination rate index (GRI) (F = 7.99, *p* < 0.001). There was no significant difference in final germination and GRI between husked, dehusked-membraned and dehusked membraned plus detached husk. However, soaking husked grains in water for 24 h resulted in a significant increase in both final germination and GRI, as compared to all husk treatments (i.e., husked, dehusked-demembraned and dehusked-membraned grains). Final germination for soaked and non-soaked husked grains was 91.7% and 70%, respectively. Similarly, GRI for soaked and non-soaked husked grains was 47.7 and 43.1, respectively. Dehusked-membraned grains attained higher final germination and GRI than dehusked-demembraned grains. Interestingly, neither soaking nor adding detached husk to dehusked grains affected final germination or GRI (Table 1).

### 2.2. Effect of Husk, Light and Temperature on Final Germination

**Figure 2:**
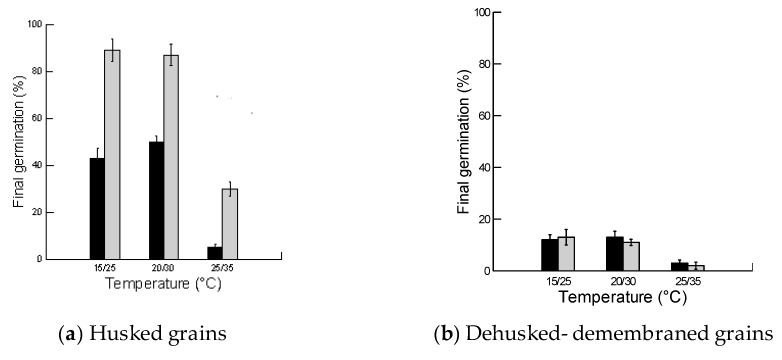
Effects of light and temperature of the incubation on final germination percentage (mean ± SE) of husked and dehusked-demembraned grains of *Brachypodium hybridum*. The grains were soaked for 24 h before germination in three programmed growth chambers. Dark and light bars are for dark and light germination, respectively. The mean of each treatment is from four replicate dishes, each with 20 seeds.

Three-way ANOVA demonstrated the significant impact of the husk, light and temperature of grain incubation and all of their interactions on final germination of *B. hybridum* grains (*p* < 0.01, Table 2). Husked grains attained significantly greater overall germination than dehusked-demembraned grains. In addition, overall germination was significantly greater under light and at lower and moderate temperatures in comparison to darkness and higher temperatures (Figure 2).

Husked grains attained significantly greater germination under lighted conditions rather than darkness. At lower and moderate temperatures (15/25 °C and 20/30 °C), germination of husked grains reached around 90% in light, but only up to 43% and 45% respectively under darkness. At higher temperatures (25/35 °C), germination of husked grains was significantly reduced to 30% under light and 5% in darkness. The overall results indicate that germination in darkness was reduced at all temperatures, but the reduction was more pronounced at higher temperatures (Figure 2a). In dehusked-demembraned grains, germination was significantly reduced at all temperatures, and almost completely inhibited at 25/35 °C. However, there was no significant difference between germination in light and darkness respectively at all temperatures. The germination of dehusked- demembraned grains ranged between 11% and 13% under light and darkness at both low as well as high temperatures (Figure 2b).

### 2.3. Effect of Husk and Temperature on Germination Rate Index

**Figure 3:**
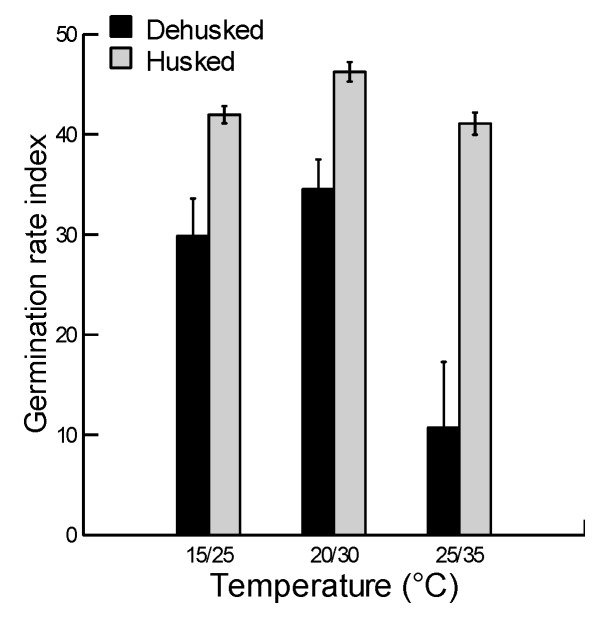
Effects of incubation temperature on germination rate index (mean ± SE) of husked and dehusked-demembraned grains of *Brachypodium hybridum*. The grains were soaked for 24 hrs before germination in three programmed growth chambers. The mean of each treatment is from four replicate dishes, each with 20 seeds.

Results of two-way ANOVA demonstrated the significant effects of the husk, temperature of seed incubation and their interaction on the GRI of *B. hybridum* (*p* < 0.001, Table 2). Similar to final germination, GRI was greater in husked compared to dehusked-demembraned grains and the difference was more obvious at the highest temperatures (Figure 3). The reduction of GRI observed in dehusked-demembraned grains was also more pronounced at higher temperatures. The overall results indicate that the germination speed of husked grains remained high and was not affected by temperature, and that of dehusked-demembraned grains was low with the reduction greater at higher temperatures.

### 2.4. Effect of Dormancy Regulating Compounds

**Figure 4:**
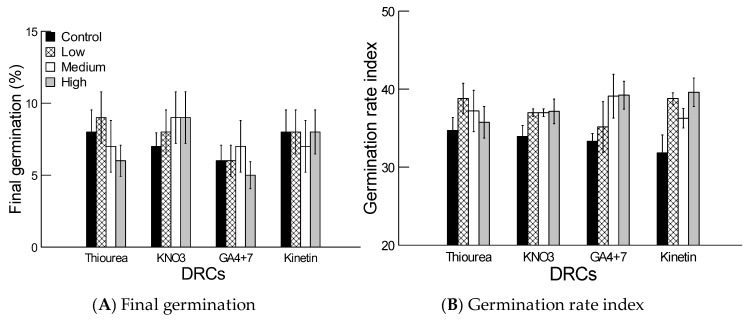
Effect of different concentrations of different dormancy regulating compounds (DRCs) on (**A**) final germination percentage and (**B**) germination rate index (mean ± SE) of dehusked-demembraned grains of *Brachypodium hybridum*. Low, medium and high concentrations were 5, 10 and 15 mM for thiourea, KNO_3_, 0.25, 0.5 and 0.75 mM for GA_4+7_ and 0.05, 0.25 and 0.5 mM for Kinetin, respectively.

There were no significant effects of dormancy regulating compounds (DRCs) at various concentrations on final germination and GRI (*p* > 0.05, Table 3) of dehusked-demembraned grains. None of three different concentrations in each of the four DRCs was able to enhance final germination or germination speed in comparison to the control (Figure 4A,B). However, the interaction of DRCs and their concentrations was significant on GRI (*p* < 0.01), but not on the final germination (*p* > 0.05, Table 3). Amongst the different DRCs, the GRI was significantly higher in at least one concentration in comparison to the control (Figure 4B).

### 2.5. Effect of Light Quality

**Figure 5:**
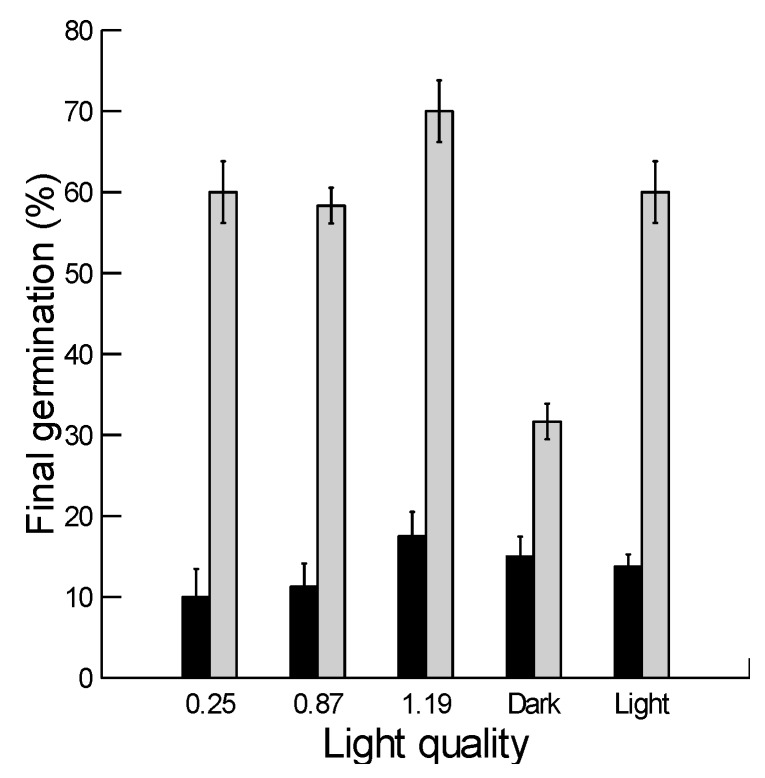
Effect of different light qualities on final germination percentage (mean ± SE) of dehusked-demembraned (dark bars) and dehusked-membraned (light bars) grains of *Brachypodium hybridum.*

Significant effects were observed for both husk (F = 755, *p* < 0.001) and light quality (F = 20.5, *p* < 0.001) as well as their interactions (F = 19.86, *p* < 0.001) on the final germination of *B. hybridum*. Dehusked-membraned grains attained significantly greater germination at all light qualities, as compared with Dehusked-demembraned grains. There was no significant difference observed in the final germination of dehusked-demembraned grains between different light qualities. However, dehusked-membraned grains attained significantly greater germination in red-rich light (R:FR = 1.19) in comparison to other light qualities. In addition, germination of dehusked-membraned grains was significantly lower in darkness than under other light settings (Figure 5).

### 2.6. Effects of Husk Treatments on Plant Growth

#### 2.6.1. Seedling Longest Leaf in Petri Dishes

There was a highly significant effect of husk treatment on the length of largest leaf in seedlings grown under potted soil germination (F = 14.22, *p* < 0.001). Seedlings of both soaked and non-soaked husked grains had significantly longer leaves than all other husk treatments. In addition, seedlings from soaked husked grains produced significantly longer leaves (6 cm) than those from non-soaked husked grains (5.28 cm). In addition, seedlings from dehusked-membraned soaked grains were significantly longer (3.93 cm) than those from all non-soaked dehusked grains (Table 1).

#### 2.6.2. Seedling Growth in Potted Soil

The effect of husk was significant on both seedling fresh weight (F = 267, *p* < 0.001) and dry weight (F = 95.3, *p* < 0.001). Seedlings (roots and shoots) from husked grains attained significantly heavier fresh weight (16.18 ± 0.24 mg) than those from dehusked grains (9.22 ± 0.38 mg). In addition, the dry weight of seedlings from husked grains was significantly greater (1.82 ± 0.028 mg) than that of seedlings from dehusked grains (1.19 ± 0.067 mg).

## 3. Discussion

Several studies have assessed the function and significance of the presence of dead, hardened husks on grain dormancy and germination in several grass species, with inconsistent results. Whereas dead structures surrounding grains enhanced germination in some species, such as *Festuca rubra* [7] and *Lolium perenne* [6], they inhibited germination and induced dormancy in other grasses such as wild emmer wheat [4] and *B. distachyon* [12]. Our results showed insignificant differences in final germination and GRI between non-soaked husked and dehusked-membraned grains. However, husked grains produced longer leaves in comparison to dehusked grains (Table 1). This effect is not consistent with the impact of husk in *B. distachyon*, a close relative of *B. hybridum*, in which the presence of husk inhibited germination (especially under lighted conditions) and the manual removal of husks significantly increased germination [12]. However, our finding is consistent with another species of *Brachypodium* (*B. rupestre*) in which there was no significant difference in final germination between husked and dehusked seeds [42]. In *B. rupestre*, however, dehusked grains had a higher speed of germination but not a significantly different germination percentage in comparison to husked grains [42]. The inconsistency observed in the role of husks on germination in *B. distachyon* and *B. hybridum* respectively could be attributed to the maternal environment during seed development and maturation. Whereas *B. distachyon* is a summer annual in temperate climate at the northern latitudes [12], *B. hybridum* is a winter annual in the arid hot climate of the Arabian desert [20]. Environmental conditions can affect the chemical compositions of different parts of grasses, such as *Lolium perenne* [43]. Further biochemical and physiological investigations are needed to understand the role of husks in regulating the germination process in different species of the *Brachypodium* species complex.

Our results showed significantly greater germination under light than in darkness in husked grains. The lower germination in darkness was more pronounced at higher temperatures. In dehusked-demembraned grains, however, there was no significant difference between germination in light and darkness at all temperatures (Figure 2b). In the genetic model *B. distachyon*, Barrero et al. [12] indicated that germination was significantly greater in dark than in light for both husked and dehusked fresh harvested grains. After 16 weeks of after-ripening, dehusked grains fully germinated in both light and darkness, while husked grains fully germinated in the dark and displayed only 20% germination in light [12]. The difference in husk effects between the two *Brachypodium* species (*B. hybridum* and *B. distachyon*) could be genetic or due to the maternal environment under which seeds were developed and matured. As the former is a winter annual in the arid UAE, its grains are matured under shorter days. In contrast, *B. distachyon* is a summer annual at higher latitudes with grain maturation occurring under longer days. In several species, day length has been observed to affect light requirement during germination [15,18,22,44,45].

Light requirement during seed germination could be determined through a group of active forms of phytochromes, which are a class of photoreceptors that persist in dry seeds. It has been proposed that tissues surrounding the embryo can filter the white light spectrum to specific wavelengths, which can regulate specific phytochromes that modulate the seed germination process [22,46]. Our results showed that germination of dehusked-membraned grains was significantly greater in red-rich light (R:FR ratio of 1.19). Conversely, germination was observed as significantly lower in darkness and other light settings (white light, R: FR ratios of 0.25 and 0.87) (Figure 5). In a previous study, we found that germination of husked grains had a similar response to different light settings; i.e., they germinated more under red-rich light and less in darkness [20]. In dehusked-demembraned grains, however, there was no significant difference in the final germination triggered under different light qualities (Figure 5). This indicates that the inner membrane could be partially responsible for dormancy regulation. Filtration of red-rich light (F:FR = 1.19) allowed greater germination, as compared with far rich light (R:FR ratio of 0.25). A similar role for inner membranes was reported in on germination response of *Citrullus colocynthis* seeds, where continuous light greatly reduced final germination while removal of the inner membrane (exocarp) resulted in stimulation of light-dependent germination [38]. Another possible role for the inner membrane in seed germination could be through controlling the passage of imbibed water. For example, it has been observed that the thin inner membranous layer acted as an effective barrier against water imbibition and hence reduced germination in *Pinus lambertiana* [47] and *P. monticola* [48].

Several studies have reported the importance of phytohormones (such as abscisic acid, gibberellin and cytokinins) and nitrogenous compounds (such as nitrate and thiourea) in regulating seed dormancy and the germination process [27]. Exogenous application of these DRCs alleviated innate dormancy and enhanced final germination and the germination rate of several grasses in arid and hyper-arid deserts [21,24,25,26]. Our results indicated that treatment of dehusked demembraned grains with different concentrations of various DRCs did not increase germination in comparison to non-treated grains (Figure 4). This would indicate that reduction in final germination of the dehusked-demembraned grains cannot be explained in light of the utilized phytohormones and nitrogenous compounds. Studies have shown that DRCs did not alleviate innate dormancy in grains of other grasses found in the arid tropical climate of Arabia such as *Coelachyrum brevifolium* and *Pennisetum divisum* [21], *Sporobolus arabicus* [24], *Panicum turgidum* [25], as well as *Aeluropus lagopoides*, *Halopyrum mucronatum* and *Sporobolus ioclados* [49].

Our results showed that growth was significantly greater in *B. hybridum* seedlings from husked grains in comparison to those derived from dehusked grains. Similarly, *Eurotia lanata*, vigor and radical growth of seedlings derived from seeds covered with hairy bract surrounding the structures demonstrated almost double the amount of seedlings in comparison to threshed seeds [50]. In addition, seedlings grown from the intact dispersal unit of wild emmer wheat attained significantly greater growth in comparison to those derived from naked fruits [4]. The authors attributed this effect to the release of growth-promoting substances from dead floral bracts. These dead structures function as long-term storage for hundreds of proteins that are released upon hydration to assist in food hydrolysis and detoxification of reactive oxygen species and nutrients [3,51].

Our results indicated that soaked husked seeds attained greater and faster germination as well as faster seedling growth in comparison to non-soaked husked seeds. This may be attributed to a possible hydropriming effect of the soaking process and/or release of certain germination deterrents that may be present in the husks. During hydropriming, seeds imbibe water and undergo the earlier stages of germination in which pre-germination metabolic activities are preceded while the latter stages of germination are inhibited [52,53]. Our results also indicate that soaking the dehusked-membraned and dehusked–demembraned grains resulted in significant increases in seedling growth in comparison to non-soaked dehusked-membraned and dehusked–demembraned grains. This would further support the postulated positive hydropriming effect on seedling growth. In addition, as the positive effect of grain soaking on final germination and GRI was significant only for husked rather than soaked dehusked-membraned and soaked dehusked-demembraned grains, leaching certain germination inhibitors could be a possible explanation for this effect. Wurzburger and Leshem et al. [9] attributed germination inhibition observed in the grass *Aegilops kotschyi* to the production of germination inhibitors such as coumarin or abscising that were released from the glume and hull upon fruit soaking [4].

## 4. Material and methods

### 4.1. Seed Collection and Preparation

Mature spikes of *B. hybridum* were collected during March 2016 from Wadi Helo (24°56’31.12” Latitude and: 56°12’1.34” Longitude), which runs parallel to the east coast of the United Arab Emirates (UAE). The spikes were collected from shaded gorges situated at 700 to 1000 m above sea level in the north-facing aspect of Wadi Helo, which is the typical microhabitat of *B. hybridum* in the arid mountains of the Arabia [54]. Seeds were randomly collected from more than 400 individuals to cover the genetic diversity of the whole population. Some spikes were air-dried and stored in brown paper bags for after-ripening for two years at room temperatures (22 ± 2 °C, hereafter referred to as husked grains). Other spikes were manually dehusked. The inner membrane of the dehusked grains was either kept intact (hereafter referred to as dehusked-membraned grains) or removed (hereafter referred to as dehusked-demembraned grains).

### 4.2. Effects of Husk Treatments on Germination and Seedling Growth in Petri Dishes

In order to assess the impact of the presence of the husk and/or inner membrane on germination and seedling growth of *B. hybridum*, different husk, membrane and soaking treatments were conducted in a petri dish experiment. The utilized treatments were (a) husked grains, (b) husked grains soaked for 24 h, (c) dehusked-membraned grains, (d) dehusked-membraned grains + detached husk, (e) dehusked-membraned grains soaked for 24 h, (f) dehusked-demembraned, (g) dehusked-demembraned + detached husk, (h) dehusked-demembraned soaked for 24 h. As soaking could be considered as a hydropriming process that might enhance germination level, speed and hence seedling growth [52,53], soaking was conducted for husked, dehusked-membraned and dehusked-demembraned grains.

Germination was conducted in 9-cm Petri dishes containing a filter paper with 10 mL of distilled water. Four replicate dishes, each with 20 seeds, were utilized for each treatment. The dishes were incubated in programmed growth chambers adjusted at 12 h dark/12 h light cycles in 15/25 °C. The provided light was cool white fluorescent light with an intensity of 960-µmol m^−2^ s^−1^. A seed was considered to be germinated following radical emergence. Emerged seedlings were moved to other Petri dishes and misted daily. After 15 days of radical emergence, the longest leaf was measured for every seedling.

### 4.3. Effect of Husk, Light and Temperature on Final Germination

Husked and dehusked-demembraned grains were soaked for 24 h and germinated in three programmed growth chambers adjusted at 12 h dark/12 h light cycles in 15/25°C, 20/30°C and 25/35°C in both continuous darkness and alternating dark and light. Petri dishes were wrapped with aluminum foil to achieve dark conditions. Germination was conducted in 9-cm Petri dishes containing a filter paper with 10 mL of distilled water. Four replicate dishes, each with 20 seeds, were utilized for each treatment. A seed was considered to be germinated following radical emergence. Emerged seedlings were counted and removed every other day for a total of 16 days after seed soaking. Seeds incubated in the dark were checked only once at the end of the experiment (i.e., after 16 days).

### 4.4. Effect of Dormancy Regulating Compounds

In order to assess the role of structures surrounding the grains in the production of dormancy regulating compounds (DRC), the effects of exogenous application of five DRCs were assessed on final germination of dehusked-demembraned grains. The utilized DRCs and their concentrations were: thiourea (5, 10 and 15 mM), nitrate (KNO_3_, 5, 10 and 15 mM), gibberellic acid (GA_4+7_, 0.25, 0.5 and 0.75 mM) and kinetin (0.05, 0.25 and 0.5 mM). These concentrations were selected based on the results of similar previous studies assessing the impact of the utilized DRCs on seed germination in other grasses found in the subtropical Arabian Desert [21,25,26]. Germination was conducted as above in an incubator adjusted at daily 12 h dark/12 h light cycles in 15/25 °C.

### 4.5. Effect of Light Quality

Dehusked-demembraned and dehusked-membraned grains of *B. hybridum* were germinated in five light treatments: continuous darkness, alternating 12 h dark/12 h light and three filters (Lee Filter, London, UK) that created a gradient in the R: FR ratio (0.25 –1.19) by filtering cool white fluorescent light. The intensity of cool white fluorescent light was 960-µmol m^−2^ s^−1^. We used the filters (Lee Filter, London, UK) to obtain different R (650–670 nm): FR (720–740) ratios. Filters 245, 088 and 089+245 were used to produce R:FR ratios of 1.19, 0.87 and 0.25. The utilized light qualities were verified by measuring R:FR light ratios in germination trays covered with different filters with an SKR110 red/far-red sensor (Skye Instruments, Powys, UK). The germination was conducted as above in an incubator adjusted at daily 12 h dark/12 h light cycles in 15/25 °C.

### 4.6. Effects of Husk Treatments on Seedling Growth in a Pot Experiment

In order to assess the impact of the presence of husk and/or inner membrane on seedling growth of *B. hybridum*, husked and dehusked-demembraned grains were sown at 0.2 cm depth in 10-cm diameter pots filled with a soil mix of sand, peat moss and perlite in a 1:1:1 ratio. Five replicate pots were used for each treatment. Each pot had 15 grains for husked and 35 grains for dehusked-demembraned treatments; germination of the husked grains was found to be higher than dehusked grains. Pots were put in a programed growth chamber adjusted at daily 12 h dark/12 h light cycles in 15/25 °C. The seedling density was kept at five per pot after 7 days post-plumule emergence. Pots were watered every other day to keep soil moist. After 18 days, seedlings were harvested and root and shoot lengths were measured to the nearest millimeter. In addition, fresh weight was assessed immediately after harvest. Dry weight was assessed after drying the seedlings for 4 days at 70 °C, when the seedling weight was constant for two successive days. For statistical analysis, the average of five seedlings per pot was used as a replicate.

### 4.7. Data Analyses

Germination rate index (GRI) was calculated using a modified Timson’s germination velocity index [55]. Three-way ANOVA was used to assess the significance of three main factors (husk treatment, thermoperiod and photoperiod during germination) and their interactions on final germination of *B. hybridum* seeds. Two-way ANOVA was used to assess the effect of husk treatment and thermoperiod during germination on GRI. In addition, two-way ANOVA was used to assess the effect of husk treatment (dehusked-demembraned and dehusked-membraned grains) and light quality on final germination of *B. hybridum*. One-way ANOVA was used to assess the significance of the difference between the different husk treatments (soaked husked, dehusked-membraned, dehusked-membraned + detached husk, soaked dehusked-membraned, dehusked-demembraned, dehusked-demembraned + detached husk, soaked dehusked-demembraned) on final germination, GRI and length of the largest leaf. The same test was used to assess the effect of DRCs and light quality on final germination. Tukey’s tests were performed to test pair-wise differences between means. The final germination percentages and GRI were arcsine- and log-transformed, respectively, to meet the assumptions of ANOVA. This transformation improved the normality of the distribution of the data. ANOVA and Tukey’s tests were performed by using the General Linear Model (GLM) procedure of SYSTAT, version 13.0.

## 5. Conclusions

The presence of husks in non-soaked seeds had a neutral effect on germination level and rate, but a positive effect on seedling growth. The inner membrane could be partially responsible for dormancy regulation through filtering light qualities with specific wavelengths. Further studies are required to assess the possible germination and seedling growth roles of the inner membrane around *B. hybridum* grains. For economic considerations of *B. hybridum* as a forage and cover crop, soaked husked grains should be seeded.

## Figures and Tables

**Table 1:** Effect of husk treatments on final germination in light (FG), germination rate index (GRI) and longest leaves (mean ± SE) of *Brachypodium hybridum* grains and seedlings. Means of different husk treatments having the same letters within each variable are not significantly different at *p* ≤ 0.05.

Husk Treatments	FG%	GRI	Longest Leaf (cm)
Husked	70.0 ± 4.3 ^b^	43.1 ± 1.4 ^b^	5.28 ± 0.67 ^b^
Soaked husked	91.7 ± 3.2 ^a^	47.7 ± 0.8 ^a^	6.00 ± 0.15 ^a^
Dehusked-membraned	64.3 ±2.4 ^b^	43.8 ± 1.0 ^b^	2.53 ± 0.19 ^d^
Dehusked-membraned + detached husk	63.4 ± 3.5 ^b^	41.7 ± 3.9 ^b^	2.33 ± 0.17 ^d^
Soaked dehusked-membraned	63.8 ± 3.4 ^b^	44.0 ± 1.7 ^b^	3.93 ± 0.28 ^c^
Dehusked-demembraned	8.3 ± 1.7 ^c^	28.5 ± 2.9 ^c^	1.83 ± 0.44 ^d^
Dehusked-demembraned + detached husk	8.3 ± 1.7 ^c^	31.3 ± 2.6 ^c^	1.88 ± 0.36 ^d^
Soaked dehusked-demembraned	10.0 ± 1.9 ^c^	31.3 ± 2.6 ^c^	3.55 ± 0.24 ^c^
F-value, P (One way ANOVA)	58.5, *p* < 0.001	7.99, *p* < 0.001	14.22, *p* < 0.001

**Table 2:** Results of ANOVA showing the effects of husk treatment as well as light and temperature of the grain incubation on final germination, and the effect of husk treatment and temperature on the germination rate index of *Brachypodium hybridum*.

Source of Variation	Df	Mean Squares	F-Ratio	*p*-Value
**(a) Final germination**				
Husk	1	2.953	475.794	<0.001
Temperature (Temp)	2	0.674	108.626	<0.001
Light	1	0.709	114.277	<0.001
Husk × Temp	2	0.354	57.095	<0.001
Husk × Light	1	0.749	120.641	<0.001
Temp × Light	2	0.050	8.125	<0.01
Husk × Temp*Light	2	0.044	7.086	<0.01
Error	36	0.006		
**(b) Germination rate index**				
Husk	1	2.416	88.575	<0.001
Temperature (Temp)	2	0.770	28.209	<0.001
Husk × Temp	2	0.579	21.236	<0.001
Error	16	0.027		

**Table 3:** Results of ANOVA showing the effects of dormancy regulating compounds (DRCs) at various concentrations on final germination and the effect of husk treatment and temperature on the germination rate index of *Brachypodium hybridum*. ns: insignificant difference at *p* ≤ 0.05.

Source of Variation.	Df	Mean Squares	F-Ratio	*p*-Value
**(a) Final germination**				
DRCs	3	0.002	1.452	ns
Concentration (C)	3	0.000	0.161	ns
DRCs × C	9	0.000	0.398	ns
Error	48	0.001		
**(b) Germination rate index**				
DRCs	3	0.000	0.008	ns
Concentration (C)	3	0.054	4.189	<0.01
DRCs × C	9	0.010	0.769	ns
Error	48	0.013		
